# Moribund Ants Do Not Call for Help

**DOI:** 10.1371/journal.pone.0151925

**Published:** 2016-03-17

**Authors:** Krzysztof Miler

**Affiliations:** Institute of Environmental Sciences, Jagiellonian University, Kraków, Poland; University of Paris 13, FRANCE

## Abstract

When an antlion captures a foraging ant, the victim’s nestmates may display rescue behaviour. This study tested the hypothesis that the expression of rescue behaviour depends on the life expectancy of the captured ant. This hypothesis predicts that the expression of rescue behaviour will be less frequent when the captured ant has a lower life expectancy than when it has a higher life expectancy because such a response would be adaptive at the colony level. Indeed, significant differences were found in the frequency of rescue behaviours in response to antlion victims with differing life expectancies. In agreement with prediction, victims with lower life expectancies were rescued less frequently, and those rescues had a longer latency and shorter duration. There was also a qualitative difference in the behaviour of rescuers to victims from the low and high life expectancy groups. Several explanations for these findings are proposed.

## Introduction

Foraging ants are faced with a serious threat from co-occurring trap-building antlion larvae because these predators are highly specialized for capturing terrestrial invertebrates [1]. Some ant species have evolved means of avoiding antlion predation, i.e., avoiding areas where antlions form aggregations [2]. Nevertheless, in the event that an ant is captured by an antlion larva, nearby nestmates may exhibit risky rescue behaviour to save the captured ant from predation [3]. This behaviour can be displayed by one or more individuals (rescuers) and is directed towards another individual (victim) to allow the latter to free itself from a dangerous situation [4]. In the context of antlion capture, rescue behaviour is possible because the steps required for the larva to firmly grip and begin consuming the ant take some time. Rescue of the captured ant can take several forms spanning several behavioural categories, from relatively simple digging around the victim and pulling on the victim’s limbs to more precise behaviours such as removing sand that is covering the victim and directly attacking the mandibles of the antlion [3,5]. When engaged in a rescue attempt, the rescuer faces a serious threat of becoming a victim itself, which is why this type of behaviour has been considered highly altruistic [4]. Although the mechanism underlying the rescue behaviour remains a mystery, it is hypothesized that it is mediated by the action of the captured ant, which releases a facilitating pheromone. Observations made by Czechowski and co-authors strongly suggest that ants in distress do emit signals that summon their nestmates [3]. These signals are colony-specific [6] and may originate from the Dufour’s and poison glands, at least in some ant species [7]. Furthermore, the fact that anesthetized ants elicit no rescue [6,7] strongly supports the notion that active pheromone release by the victim is necessary for the expression of rescue behaviour. Therefore, the behaviour of rescuers may be considered as an indicator of the behaviour of the ant in distress, at least with respect to call for help signalling.

The present study tested the hypothesis that the frequency of expression and characteristics of rescue behaviour exhibited by rescuer ants depend on the life expectancy of the victim ants. Such a relationship between rescue behaviour and life expectancy may be adaptive for two nonexclusive reasons. First, this relationship may constitute a broader social isolation syndrome of moribund ants. Specifically, dying ants serve their inclusive fitness by leaving their nests [8]. Their own perceived low life expectancy serves as a trigger to do so, lowering their chance of interaction with nestmates. It is adaptive for individuals with low life expectancy to avoid interactions, because such individuals are typically sick, and may easily transmit disease within the nest [9]. Rescue behaviour is a form of interaction between nestmates, and as with any other type of interaction, it can be avoided by moribund ants. Second, the rescue behaviour and life expectancy relationship may reflect the high colony-level cost to saving individuals with low life expectancy [10]. The “division of labour by division of risk” hypothesis states that mean worker longevity is prolonged and overall colony performance increases when safe tasks are performed by high-value workers with high life expectancy and when risky tasks are carried out by low-value workers with low life expectancy because the future benefit to the colony from soon-to-die workers is low [11–14]. Thus, in the context of rescue behaviour, investing resources into saving individuals of low value that are characterized by low life expectancy appears counter-adaptive because they are of limited use to the colony.

In agreement with both of the above explanations, it is shown here that ants more frequently rescue their nestmates and perform more intensive rescues when the imperilled individuals have a higher life expectancy than when they have a lower life expectancy. Although alternative explanations are possible, the results of this study may be interpreted as a decline in the calling for help with decreasing life expectancy in ant victims.

## Materials and Methods

Approximately 500 active *Formica cinerea* foragers were hand-picked from each of the three different colonies from the same site (Błędowska Desert). Overall, 300 antlion larvae (*Myrmeleon bore*) from the same locality were also collected. In the laboratory, antlions were kept in plastic cups half filled with dry sand (7 cm in diameter, 15 cm high), while ants from each colony were kept in separate plastic boxes (25 x 17 x 10 cm), at a constant 24°C and 40–60% RH and a 12:12 L:D cycle. Ants were fed sucrose solution ad libitum, while antlions were not provided with any food; both were allowed to habituate to the setup for two days after transportation from the field. Then, on day three, in each ant colony, four groups of workers were created (50 ants each) and kept in separate plastic boxes (25 x 17 x 10 cm), while the rest (approximately 300 per colony) remained in their original plastic boxes. Two of these groups were untreated controls, and the other two were experimental groups whose life expectancy was artificially shortened by exposure to ~100% carbon dioxide for 1.5 h. The method used for the experimental manipulation has been used previously, and its efficiency in lifespan shortening in ants is well established [15,16]. One control and one experimental group were used as the sources of the captured ants during the tests, and the other two groups of workers were used to establish that the carbon dioxide treatment was successful (checked daily until all were dead). For the tests, which were conducted on the day after the carbon dioxide treatment, an antlion larvae capture bioassay was performed inside the cups in which the antlions were kept. In each test, a control forager or a forager with shortened life expectancy was dropped into the antlion pit. Immediately after the ant was captured, a potential rescuer (one of the remaining control ants from the respective colony) was introduced into the cup but not into the pit. Each test lasted three minutes, during which it was noted whether rescue behaviour occurred, and if so, the latency to the first episode of rescue, the total duration of rescue, and the types of behavioural categories displayed by the rescuer. Four behavioural categories of rescue were used: pulling at the victim’s limbs/antennae/mandibles, digging around the victim, removal of sand covering the victim, and direct attack on the antlion mandibles. The operational definitions of behavioural categories were the same as in previous studies of ant rescue behaviour [5,6], except that the definition of pulling in the present study was applied to antennae and mandibles as well as limbs. The order of testing within each colony was counterbalanced for both the control and experimental groups. All tests that lasted less than three minutes (because the victim was completely buried under the sand or was released from the grasp of the predator for some reason) were excluded. The final number of tests in each group from each colony was 30. No ants or antlions were used more than once. Data were analysed in SPSS Statistics 21 software (IBM, Warsaw, Poland). The two-tailed Fisher’s Exact Test (FET) was used to detect between-group differences in the occurrence of rescue, pulling, digging, sand removal and attack on the antlion. Data on mortality, the latency to and the duration of rescue were analysed with Generalized Linear Mixed Models (GLMM) using a loglink function and Poisson error distribution. Colony was included as a random factor, while group was used as a fixed factor.

## Results and Discussion

**Fig 1:**
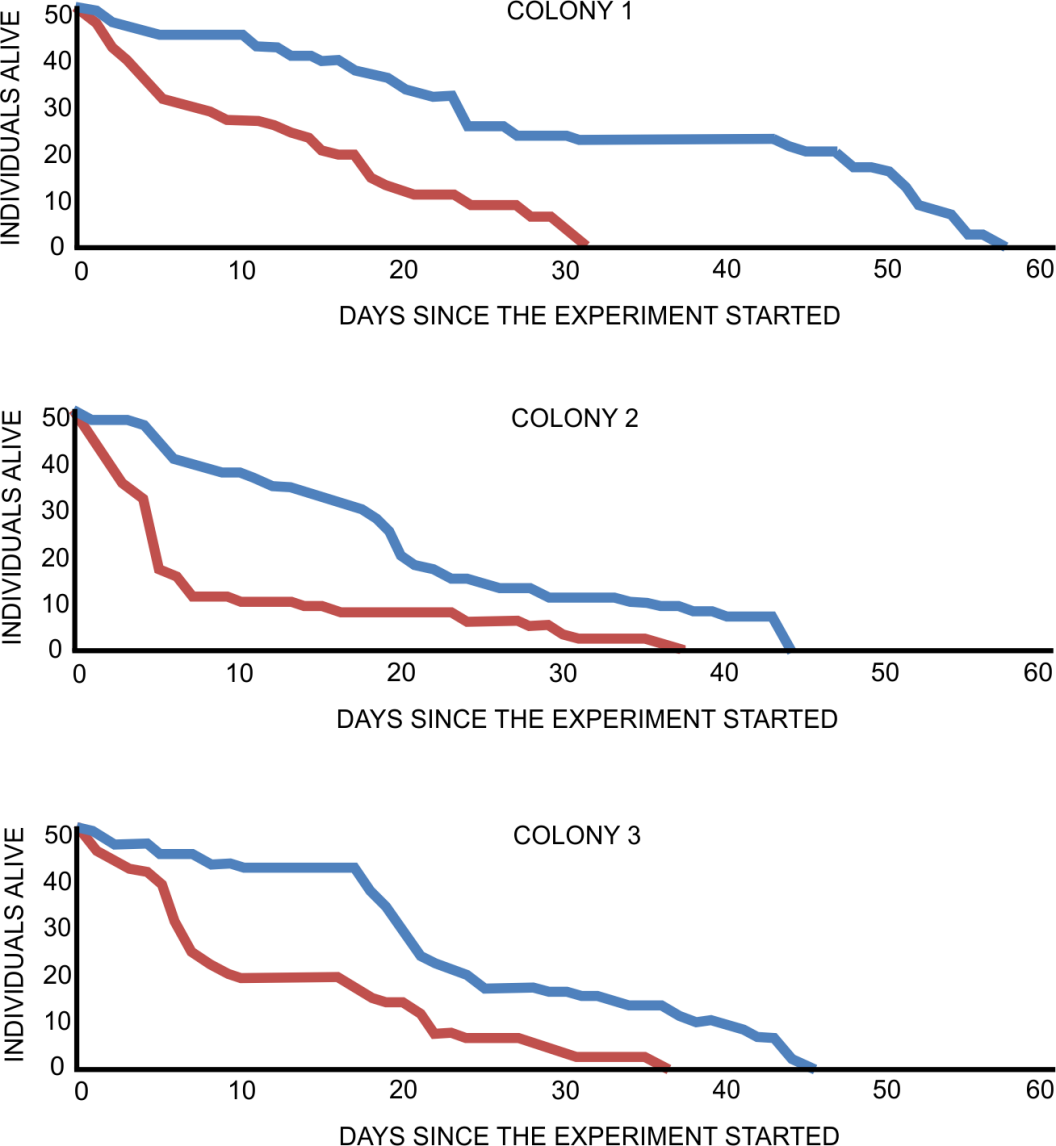
Mortality of *Formica cinerea* foragers in control and experimental treatments. Blue lines indicate untreated control groups. Red lines indicate groups with artificially shortened life expectancy induced by carbon dioxide exposure. Mortality data were analysed using GLMM. Significant differences were observed among groups (F_1,298_ = 799.697, p < 0.0001).

**Fig 2:**
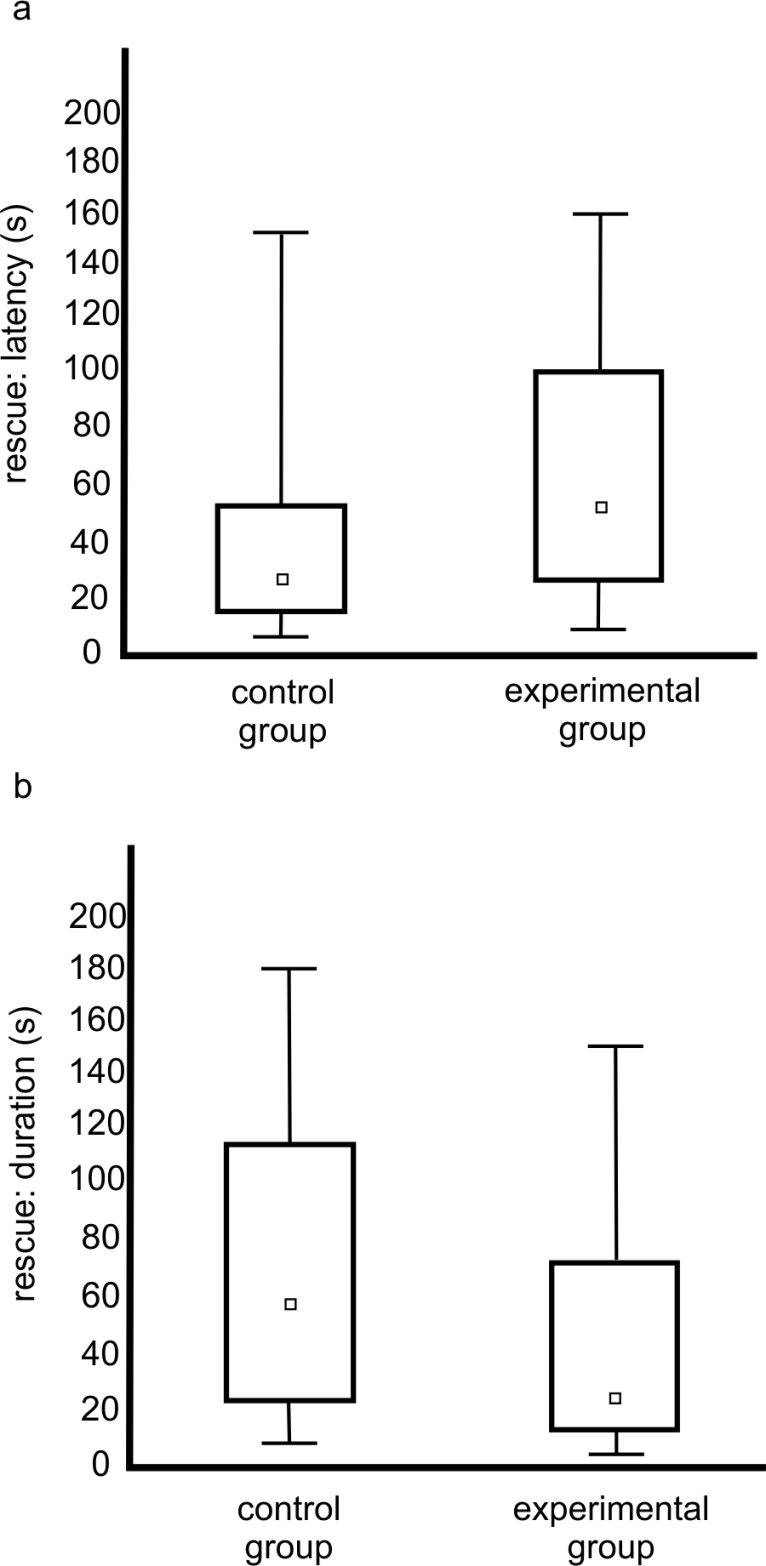
Between-group differences in rescue behaviour characteristics. The latency to rescue is presented in panel a) and the duration of rescue in panel b). Squares indicate the median, boxes indicate quartiles, and whiskers indicate the minimum and maximum values. Control groups were untreated, whereas experimental groups were exposed to carbon dioxide. Data were analysed using GLMM. Significant group differences were observed for both latency (F_1,82_ = 195.672, p < 0.0001) and duration (F_1,82_ = 218.937, p < 0.0001).

**Fig 3:**
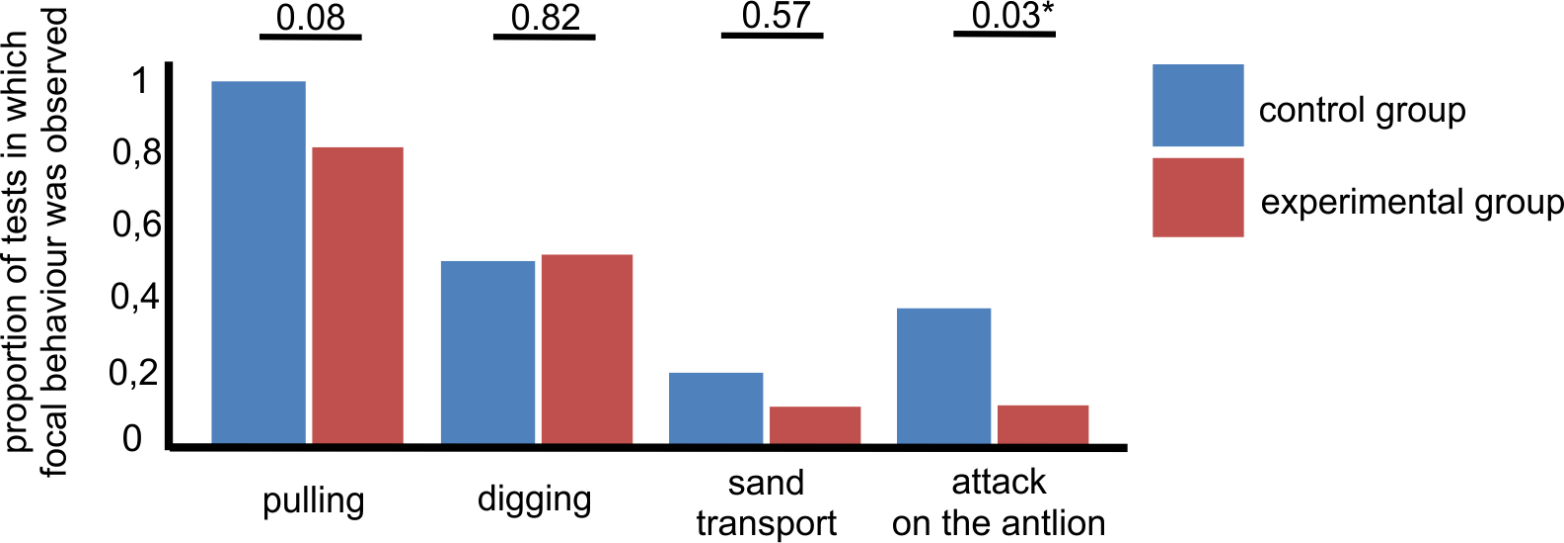
The proportion of tests in which rescue behavioural categories in control and experimental treatments were observed. Control groups were untreated, whereas experimental groups were exposed to carbon dioxide. Star indicates significance with p values for between-group differences indicated above the bars. The data were analysed separately for each behavioural category using Fisher’s Exact Tests.

The present study tested the hypothesis that the frequency of the expression of rescue behaviour is lower when the victim ants have a lower life expectancy than when they have a higher life expectancy. Life expectancy was experimentally shortened in this study using carbon dioxide exposure; this manipulation was effective at reducing life expectancy (F_1,298_ = 799.697, p < 0.0001; Fig 1). Exposure to CO_2_ is known to mimic accelerated ageing in insects [17] and may be used to study the active responses of ants to low life expectancy [8,13,15,16]. Importantly, ants of varying, unknown age and characterized by high initial variance in life expectancy were used here to establish both the untreated control and experimental (poisoned with carbon dioxide) groups of workers. As indicated by a rather high mortality in control groups, individuals with lower life expectancy were also present in the controls. This was to be expected because the ants used in this experiment were active foragers collected from the field. However, the results of the present study indicate that rescue was attempted less frequently towards ants with lower life expectancies (rescue occurred in 34 out of 90 cases in the experimental group, compared to 50 out of 90 cases in the control group; FET yielded p = 0.02). If rescue behaviour was directed towards ants with lower life expectancies, it was expressed after longer periods (F_1,82_ = 195.672, p < 0.0001) and for shorter durations (F_1,82_ = 218.937, p < 0.0001; Fig 2) than for ants with higher life expectancies. There was also a qualitative difference in the behaviour of rescuers when attempting to rescue an ant with lower life expectancy. These victims were rescued with less frequent attacks on the antlions, which was the most advanced form of rescue in the test type applied in this study (19 out of 50 cases in the control group, compared to 5 out of 34 in the experimental group; FET yielded p = 0.03; Fig 3). There were no between-group differences in the occurrence of other behavioural categories (p = 0.08 for pulling at the victim’s limbs/antennae/mandibles, p = 0.82 for digging around the victim, and p = 0.57 for transport of sand covering the victim; p values from FET; Fig 3). In general, the behaviour of potential rescuers following introduction into the test cup involved quick, erratic movements within the space provided followed by contact with the victim and, ultimately, rescue behaviour. Typically, rescue behaviour was expressed in less than one minute. Also, rescue behaviour often occurred discontinuously, i.e., with repeated breaks and shifts among behavioural categories. Importantly, because tests started immediately after the introduction of potential rescuers and lasted for a fixed time period, latency and duration data were linked. Therefore, an additional GLMM analysis for duration adjusted by latency was performed, and the adjusted duration data, multiplied and rounded, was obtained from the rescue duration time divided by test time minus the latency to rescue. This analysis revealed that group was a significant factor (GLMM: F_1,82_ = 60.131, p < 0.0001), meaning that lower rescue duration in an experimental group is not merely a reflection of less time being available for rescue due to higher latency. Further studies on rescue behaviour should account for this type of latency bias.

The present study showed that foragers characterized by a lower life expectancy elicited lower rescue levels than did those with a higher life expectancy. This result complements the study by Nowbahari and colleagues on the behavioural regulation of ant rescue by division of labour [7]. They showed that foragers dominated in both giving and receiving help, with nurses rescued for shorter durations and after higher latencies than foragers. This latter finding is unexpected, because nurses are generally characterized by higher life expectancies than foragers. From the perspectives of social isolation syndrome and the “division of labour by division of risk” hypothesis, nurses should receive higher levels of rescue than foragers: according to social isolation syndrome, nurses should be less isolated than foragers and therefore be subject to higher levels of interactions (in this case, rescue), whereas according to the “division of labour by division of risk” hypothesis, nurses are of higher value to the colony than are foragers and should therefore receive relatively higher levels of rescue effort. This contradiction between theory and experimental results may be related to worker physiology. The pheromone-based call for help by victims is considered of key importance for rescue elicitation in ants [3–7,18]. The physiological capability of workers to signal distress can be expected to change with maturation from low at the nurse stage to high at the forager stage. The ecological relevance of rescue is higher for foragers than for nurses [3,7]; therefore, foragers could be more physiologically equipped for giving and receiving rescue behaviour. Indeed, physiology and caste have been shown to be strongly interconnected in other aspects of behaviour [19].

It is plausible that the strong effect of group observed here is the result of the behaviour of the captured ants. Specifically, the between-group differences in the latency and duration of the rescue indicated that the effectiveness of initiating a rescue response declines as the life expectancy of the victim decreases. Obviously, this effect may increase colony fitness by minimizing high-cost/low-benefit behaviours. First, low life expectancy in nature indicates ill health; therefore, there is a risk of disease transmission, which may be mitigated by the social isolation of moribund ants [8]. The effect described here may be one component of a broader social isolation syndrome of dying ants. Here, it may be reflected in the reduced propensity of a dying ant to call for help when in a dangerous situation, resulting in mortality due to predation. There are likely other components of this syndrome in effect, such as food retention in the crop, that results in mortality due to the disrupted energy balance [20]. Second, moribund individuals seeking rescue may be a maladaptive behaviour from the perspective of the colony because of its cost: the effort required to save individuals with a low life expectancy is not worth the future value that they may provide [10–13]. In this case, a gradual loss of effectiveness in inducing rescue behaviour would benefit the colony because low-value individuals would not induce their nestmates to risk their own, more valuable lives. Although the present results support the predictions of both social isolation syndrome and the “division of labour by division of risk” hypothesis, additional research could provide further insight. For instance, progress may be made by studying the propensity of potential rescuers that differ in life expectancy to perform rescue behaviour.

Although this hypothesis requires further testing, ants with low life expectancy may altruistically withhold calls for help, presenting similarities to self-sacrificing [21] and waste-managing ants [22] described elsewhere. For example, in *Forelius* ants, which close their nest entrances each night from the outside, the individuals performing this task are probably near the end of their lives (i.e., have low life expectancy) and actively perform their last duty to their colony. Interestingly, the frequency of contact between these workers and their nestmates is reduced, simply because they are outside the nest, dying in solitude. Similarly, in the nests of leaf-cutting ants, only those individuals of low value to the colony and that are moribund participate in the dangerous task of waste management; their frequency of contact with nestmates is also low because waste-managing ants are isolated in specific nest compartments. Therefore, the relationship between life expectancy and isolation appears consistent among several ant species and behaviours.

In sum, moribund foragers elicit lower levels of help from their nestmates than do foragers that are characterized by higher life expectancy. Thus, the patterns of rescue behaviour in ants are even more complex than previously thought. Additional studies on rescue behaviour expression should focus on the characteristics of distress calls, which are likely critically important in inducing help behaviours in not only ants, but also other organisms that exhibit similar behaviours.

## Supporting Information

S1 File Mortality data for the control and experimental ant groups. (XLSX)

S2 File Raw data gathered in rescue behaviour tests. They include information on whether a rescue occurred, and if so, the latency and the duration as well as the behavioural categories.
(XLSX)
